# Endogenous GABA controls oligodendrocyte lineage cell number, myelination, and CNS internode length

**DOI:** 10.1002/glia.23093

**Published:** 2016-10-31

**Authors:** Nicola B. Hamilton, Laura E. Clarke, I. Lorena Arancibia‐Carcamo, Eleni Kougioumtzidou, Moritz Matthey, Ragnhildur Káradóttir, Louise Whiteley, Linda H. Bergersen, William D. Richardson, David Attwell

**Affiliations:** ^1^ Department of Neuroscience, Pharmacology and PhysiologyUniversity College LondonLondonWC1E 6BTUnited Kingdom; ^2^ Department of Cell and Developmental Biology, Wolfson Institute for Biomedical ResearchUniversity College LondonLondonWC1E 6BTUnited Kingdom; ^3^ Wellcome Trust‐MRC Cambridge Stem Cell InstituteCambridgeCB2 1QRUnited Kingdom; ^4^ Department of Oral Biology, Brain and Muscle Energy GroupUniversity of OsloBlindernOsloN‐0317Norway; ^5^ Department of AnatomyUniversity of OsloBlindernOsloN‐0317Norway

**Keywords:** oligodendrocyte, precursor, proliferation, myelination, GABA, internode

## Abstract

Adjusting the thickness and internodal length of the myelin sheath is a mechanism for tuning the conduction velocity of axons to match computational needs. Interactions between oligodendrocyte precursor cells (OPCs) and developing axons regulate the formation of myelin around axons. We now show, using organotypic cerebral cortex slices from mice expressing eGFP in Sox10‐positive oligodendrocytes, that endogenously released GABA, acting on GABA_A_ receptors, greatly reduces the number of oligodendrocyte lineage cells. The decrease in oligodendrocyte number correlates with a reduction in the amount of myelination but also an increase in internode length, a parameter previously thought to be set by the axon diameter or to be a property intrinsic to oligodendrocytes. Importantly, while TTX block of neuronal activity had no effect on oligodendrocyte lineage cell number when applied alone, it was able to completely abolish the effect of blocking GABA_A_ receptors, suggesting that control of myelination by endogenous GABA may require a permissive factor to be released from axons. In contrast, block of AMPA/KA receptors had no effect on oligodendrocyte lineage cell number or myelination. These results imply that, during development, GABA can act as a local environmental cue to control myelination and thus influence the conduction velocity of action potentials within the CNS. GLIA 2017;65:309–321

## Introduction

By speeding action potential conduction, myelination of CNS axons by oligodendrocytes increases the brain's cognitive abilities. During development or learning, an adjustment of myelin thickness or internode length may be used to tune the conduction speed of myelinated axons (Fields, 2008; Ullén, 2009). This can promote synchronous neuronal firing (Lang and Rosenbluth, 2003; Sugihara et al., 1993), make impulse propagation time less dependent on the spatial trajectory of the axon transmitting information between areas (Salami et al., 2003), or adjust propagation delays to mediate sound localization (Ford et al., 2015; Jeffress, 1948; McAlpine and Grothe, 2003; Seidl et al., 2010). Magnetic resonance imaging (MRI) reveals changes to white matter microstructure—perhaps reflecting alterations of myelination—when human subjects learn a skilled motor task such as playing the piano (Bengtsson et al., 2005) or juggling (Scholz et al., 2009). Analogous MRI changes, accompanied by elevated myelin basic protein (MBP) expression, are observed in rats trained to grasp food pellets (Sampaio‐Baptista et al., 2013), and new myelin production is necessary for mice to become skilled wheel runners (McKenzie et al., 2014). Together, these studies suggest that adaptive myelination is a normal and essential aspect of neural plasticity.

The proliferation of oligodendrocyte precursor cells (OPCs) must be controlled in order to generate the correct number of oligodendrocytes to ensheath the length of axons requiring to be myelinated. OPC proliferation and myelination are coordinated by communication between the axons to be myelinated and the developing oligodendrocyte lineage cells. This has long been known to involve growth factors, but OPCs also receive excitatory and inhibitory synaptic input mediated by glutamate and GABA (Bergles et al., 2000; Ge et al., 2009; Lin and Bergles, 2004; Karadottir et al., 2005, 2008; Kukley et al., 2007, 2008; Ziskin et al., 2007; Zonouzi et al., 2015), suggesting that these neurotransmitters may also control oligodendrocyte development and myelination. Glutamate has been suggested to block the proliferation and lineage progression of OPCs (Gallo et al., 1996; Yuan et al., 1998), but also promotes myelin formation (Lundgaard et al., 2013; Wake et al., 2011). Endogenous GABA has been reported to have no effect on OPC development (Gallo et al., 1996; Yuan et al., 1998), but may stimulate OPC migration (Tong et al., 2009) and, by inhibiting neuronal activity, might be expected to decrease myelination (Malone et al., 2013; Sampaio‐Baptista et al., 2013). However, during hypoxia, a decrease of GABA_A_ receptor mediated signaling to OPCs increases their proliferation whilst delaying myelination (Zonouzi et al., 2015).

Here we demonstrate a strong effect of endogenous GABA release on oligodendrocyte development in cerebral cortical slices. By acting on GABA_A_ receptors, GABA almost halves the number of OPCs and mature oligodendrocytes produced. Consequently, myelin coverage of axons is decreased. Furthermore, the change in the number of OPCs produced is shown to regulate the myelin sheath internode length, which has previously been postulated to be set solely by the axon diameter (Rushton, 1951) or to be an intrinsic property of the oligodendrocytes carrying out the myelination (Bechler et al., 2015). Thus, GABA release from inhibitory interneurons can tune the conduction speed of CNS axons.

## Materials and Methods

### Organotypic Cortical Slices and Myelination Assay

**Figure 1:**
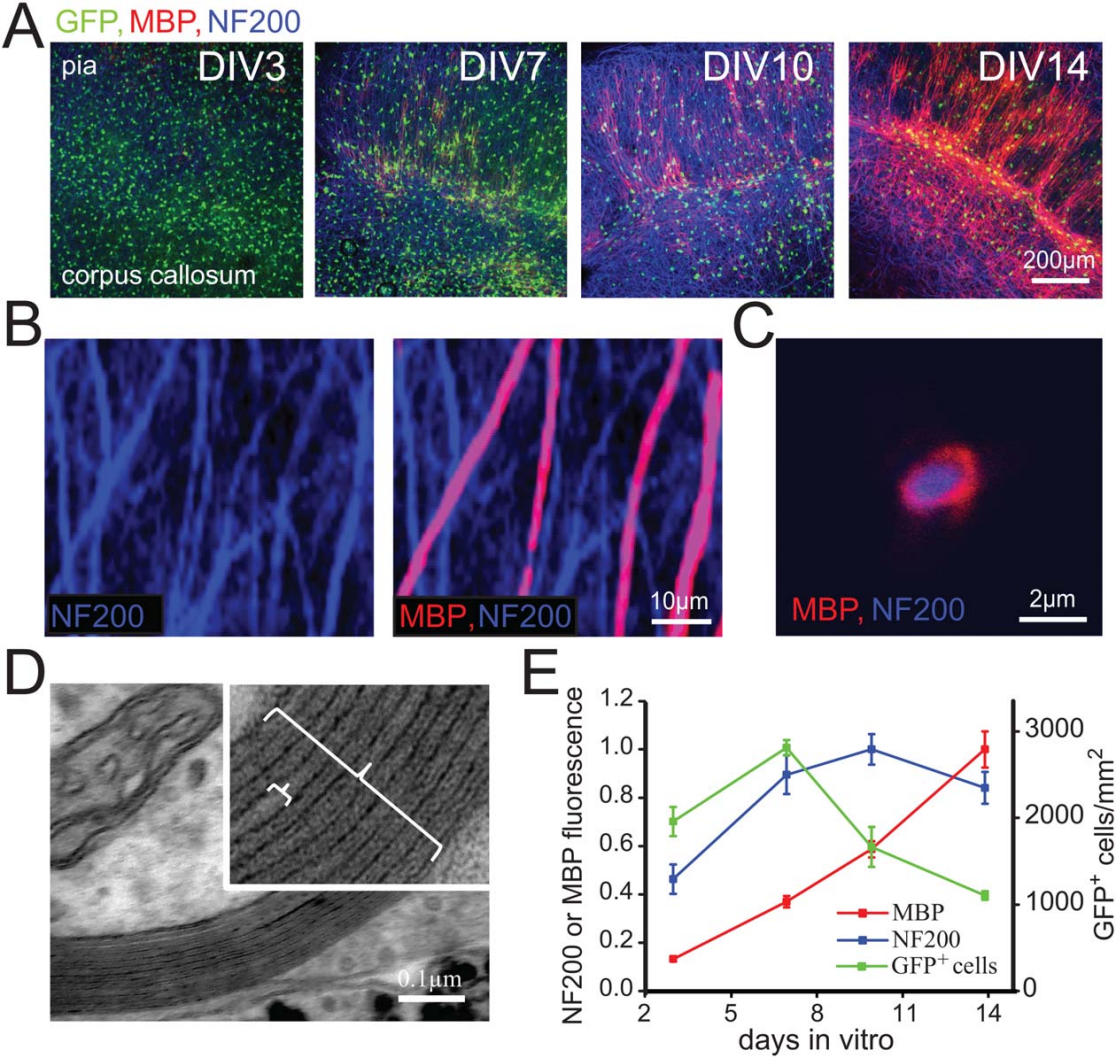
Development of myelination in cultured cortical slices. **A**: Cortical slices from mice with oligodendrocyte lineage cells expressing GFP (under control of the Sox10 promoter, green) after different numbers of days in vitro (DIV). Blue is antibody to neurofilament 200 (NF200) and shows axons; red is antibody to myelin basic protein (MBP). Slice orientation is approximately with the pial surface at the top and the corpus callosal surface at the bottom. Band of heavy myelination at DIV14 is in layers IV‐V (the Baillarger lines). **B**: Higher magnification view of cultures showing neurofilament in axons, some of which are wrapped with MBP containing myelin (green GFP channel not shown). **C**: A myelinated axon imaged in cross section. **D**: EM picture of myelin sheath (large bracket in inset) in cultured slice. The inter‐dense line distance (small bracket) was 12.55 ± 0.19 nm and the *g* ratio was 0.81 ± 0.01 in 48 sheaths. **E**: Mean values of numbers of GFP expressing cells/mm^2^ (green, right axis) and mean fluorescence (left axis) of NF200 (blue) and MBP (red) averaged over 920 μm by 920 μm images (8–15 images at different ages, using 3 animals in each of 2 experiments).

Sox10‐lox‐GFP‐STOP‐lox‐DTA (called Sox10‐GFP below) mice express GFP in oligodendrocyte lineage cells (Kessaris et al., 2006), which allowed us to develop an assay for myelination in organotypic brain slices in which oligodendrocyte lineage cells fluoresce green, and neuronal axons and myelin are identified and quantified with immunofluorescence (Fig. 1). Using this model we could assess whether changes in myelination reflected changes in the number of oligodendrocyte lineage cells, axonal density or the myelinating activity per oligodendrocyte lineage cell.

Coronal cortical slices (350 µm) from 8 day old transgenic mice were cut, and cultured (De Simoni and Yu, 2006; Rinholm et al., 2011) in medium containing 50% Minimal Essential Medium (MEM), 23% Earl's Balanced Salt Solution (EBSS), 25% horse serum, penicillin (25 units/mL) and streptomycin (25 µg/mL), all from Gibco‐Invitrogen, and 1.125% nystatin (12.5 units/mL), 36 mM glucose and 5 mM Tris base from Sigma‐Aldrich, at 37°C in a humidified atmosphere with 5% CO_2_. The extracellular concentrations of major ions were (mM) NaCl 115, NaHCO_3_ 34, NaHP0_4_ 1, KCl 5.2, CaCl_2_ 1.9, MgCl_2_ 1.1. The feeding medium was changed every 3 or 4 days. After 2 weeks the slices were fixed and immunolabeled with markers for myelin (MBP primary, and AlexaFluor 555 secondary antibodies) and axons (neurofilament (NF) 200 primary and Cy5.5 secondary antibodies: this emission is recolored blue in the figures). Images (confocal Z stacks) were taken of myelination within the gray matter of layers I–VI.

Myelination develops over about 2 weeks in these cultured slices (Fig. 1A,E). Initially many oligodendrocyte lineage cells are visible but, as neuronal processes develop, these become fewer in number as a result of cell death, and differentiate so that MBP appears. Larger magnification pictures of the cultures and electron microscope images (Fig. 1B–D) reveal that the great majority of the MBP is in compact myelin in close apposition to neurofilament‐labeled processes (only 11% of MBP labeled processes were not clearly wrapping neurofilament labeled axons, and those might be processes connecting internodes to the oligodendrocyte somata, or wrapping axons in which the neurofilament labeling was too weak to see). Thus, very few oligodendrocytes express MBP before they myelinate, and labeling is concentrated in compact myelin around axons.

By counting the number of GFP expressing cells, and using the total fluorescence of secondary antibodies to the NF antibody and to the MBP antibody as measures of the amount of neuronal processes and of myelin present (Rinholm et al., 2011), the progress of myelination in the cultures can be assessed as in Fig. 1E. During the first few days in culture the number of oligodendrocyte lineage cells increases as OPCs proliferate, which is followed by a decrease to below the initial number as cells die. After about a week the number of neuronal processes reaches a plateau, while myelination continues for at least another week. To quantify the amount of myelin per neuronal process, we calculated the ratio of the MBP fluorescence to NF fluorescence. At high magnification, in the centre of the cortex, gaps in the myelin corresponding to ankyrin G‐expressing nodes of Ranvier can be seen (Figs. 1B and 5A), allowing quantification of the number of nodes present and of internode length (Fig. 5F).

Myelination was quantified (with Metamorph or Image J) either by measuring the peak intensity of MBP labeling divided by that of the axon labeling (NF200) in the confocal stack image with the largest intensity in each stack, to obtain a measure of myelination per axon (Rinholm et al., 2011), and then averaging over 2‐6 stacks from each slice, or by counting the fraction of axons myelinated in four contiguous regions (Fig. 4I, 30 µm by 25 µm), the overall position of which was chosen randomly. The amount of myelin per length of axon was measured by placing a 3 µm by 3 µm square over the first (from the top left corner) myelinated axon found crossing the top edge of each of the regions in Fig. 4I and integrating the MBP intensity over this 3 μm square area. The internode length of the myelin sheath was measured between two nodes of Ranvier, one at least of which was within a 92 μm by 160 μm region initially imaged, and the internode was followed to its end even if that was outside the initially imaged region (Fig. 5F). These measurements of sheath length are approximately 1 μm shorter than the length that would be measured from the centre of one node to the centre of the next. Measurements of the fraction of axons myelinated, the amount of myelin per length of axon, the number of nodes of Ranvier per field of view, the length of Ranvier nodes and the length of internodes were collected with the experimenter being blind to the experimental condition. When measuring internode length, 80% of internodes remained within one 1.38 μm thick confocal image plane, while 20% extended across 2 adjacent planes in depth. The maximum error this could induce in the derived value for the internode length was 0.14%.

### Immunocytochemistry

To label organotypic slice cultures, slices were fixed in 4% paraformaldehyde in PBS for 1 h, then rinsed three times (for 10 min) in PBS followed by preincubation in 0.5% Triton and 10% goat serum in PBS for 6–8 h at room temperature. The slices were then incubated with primary antibodies for 36 h at room temperature with slight agitation, rinsed in PBS three times (for 10 min), and then incubated with secondary antibodies for 3 h before being rinsed again in PBS 3 times (for 10 min). Slices were finally mounted on a microscope slide with Citifluor (glycerol/PBS, Citifluor), covered with a 0.17 mm thick glass cover slip, and sealed with nail varnish (Boots, UK). The primary antibodies used were myelin basic protein (MBP, mouse, IgG, 1:100, Millipore, or rat, IgG 1:200, Millipore), 200kD neurofilament heavy (NF200, chicken, IgY, 1:10000 Abcam), ankyrin G (AnkG, rabbit, IgG, 1:400, Santa Cruz), APC (mouse, IgM, 1:50, Calbiochem), cleaved caspase 3 (rabbit, 1:200, Cell Signaling), and NG2 (rabbit, 1:400, Millipore). The secondary antibodies were AlexaFluor antibodies raised in goat (anti‐mouse 350, anti‐rat 555, anti‐rat 350, anti‐rabbit 568 and 633, anti‐chicken 488) or donkey (anti‐rabbit 647), all from Molecular Probes, or CY5.5 goat anti‐chicken IgY from Abcam or Jackson Labs.

### EdU Labeling and Detection

5‐ethynyl‐2′‐deoxyuridine (EdU) (Invitrogen) is a thymidine analogue that is incorporated into the DNA of cells as they undergo DNA replication (Chehrehasa et al., 2009). EdU was added to the organotypic slice medium at a concentration of 10 µM, on DIV4 for 48 h, to quantify the percentage of cells that divide between Days 4 and 6 *in vitro*. EdU developing was performed immediately following immunocytochemistry, with the Click‐iT EdU Alexa Fluor‐594 Imaging Kit (Invitrogen). Slices were immersed in Click‐iT developing cocktail and incubated for 45 min at 21ºC in the dark, according to the manufacturer's instructions. Slices were washed three times in PBS and post‐stained with DAPI (300 nM, Molecular Probes) to visualize cell nuclei.

### Electron Microscopy

At DIV14 organotypic slices were immersion fixed in 2% paraformaldehyde and 2% glutaraldehyde in 0.1 M cacodylate buffer overnight. All slices were then post‐fixed in 1% OsO_4_/0.1 M cacodylate buffer (pH 7.3) at 3°C for 2 h before washing in 0.1 M cacodylate buffer (pH 7.3). The slices were dehydrated in a graded ethanol‐water series at 3°C and infiltrated with Agar 100 resin mix. The slice was then cut perpendicularly to the plane of the slice (in the cortical region where myelination within the organotypic slice is most dense), blocked out, and hardened. Ultra‐thin sections were taken on a Reichert Ultracut S microtome. Sections were collected and stained with lead citrate. The sections were imaged using a Joel 1010 transition electron microscope and a Gatan Orius camera.

### Cortical OPC Cultures

These were as described by Lundgaard et al. (2013). Briefly, purified oligodendrocyte precursors were obtained using the shake off method of McCarthy and de Vellis (1980) applied to mixed glial cultures that had been cultured for 10 days. They were resuspended in DMEM media with modified SATO serum‐free supplement (100 µg/mL BSA, 60 ng/mL progesterone, 16.1 µg/mL putrescine, 5 ng/mL sodium selenite, 5 µg/mL insulin, 5 µg/mL *N*‐acetyl‐l‐cysteine, 50 µg/mL holo‐transferrin and 1% Pen/Strep) and growth factors (PDGF‐aa at 10 ng/mL and FGF‐b at 10 ng/mL from Peprotech; UK). OPCs were seeded at a density of 22 × 10^3^ cells/cm^2^ onto PDL coated glass coverslips yielding a purity of 86 ± 2% (*n* = 18) NG2^+^ cells after 3 days of proliferation (following the 10 days in culture with other glia).

### Electrophysiology

For studying the electrophysiology of OPCs, they were identified from their dye‐fill morphology and I‐V relations, and were whole‐cell clamped with pipettes of series resistance 5–20 MΩ. Electrode junction potentials were compensated. For experiments assessing OPC electrophysiology in organotypic slices, they were superfused at 33 ± 1^°^C with bicarbonate‐buffered solution containing (mM) 126 NaCl, 24 NaHCO_3_, 1 NaH_2_PO_4_, 2.5 KCl, 1 MgCl_2_, 2 CaCl_2_, bubbled with 95% O_2_/5% CO_2_, pH 7.4. The OPCs were voltage clamped at −64 mV and *E*_Cl_ was set to −4 mV with KCl‐based solution containing (mM) 130 KCl, 4 NaCl, 1 CaCl_2_, 10 HEPES, 10 EGTA, 4 MgATP, 0.5 Na_2_GTP, 0.05 AlexaFluor594 (pH 7.15). For experiments assessing OPC electrophysiology in culture, the cells were clamped at −44 mV and superfused at 22^°^C with HEPES‐buffered solution containing (mM): 144 NaCl, 2.5 KCl, 10 HEPES, 1 NaH_2_PO_4_,2.5 CaCl_2_, 10 glucose (pH 7.4). *E*_Cl_ was set to −87 mV by using a K‐gluconate based internal solution containing (mM): 130 K‐gluconate, 4 NaCl, 0.5 CaCl_2_, 10 HEPES, 10 BAPTA, 4 MgATP, 0.5 Na_2_GTP, 2 K‐Lucifer yellow (pH 7.3).

### Statistics

Data are shown as mean ± s.e.m. Unless stated otherwise, Student's 2‐tailed *t*‐tests were used. For multiple comparisons, *P* values were corrected using a procedure equivalent to the Holm‐Bonferroni method (for N comparisons in an experiment, the most significant *P* value is multiplied by N, the 2nd most significant by N‐1, the 3rd most significant by N‐2, etc.; corrected *P* values are considered significant if they are less than 0.05). Analysis of variance showed that most variability in the data was between different slices rather than between experiments done on different days, so when pooling data between different experiments we used the number of slices as the number of observations for statistical calculations. Numbers on figure bars show number of slices, except where stated otherwise.

## Results

### GABA, But Not Glutamate, Regulates the Number of Oligodendrocyte Lineage Cells

GABA evokes a current in OPCs (Lin and Bergles, 2004), and will also alter neuronal firing which can influence OPC proliferation and myelination (Gibson et al., 2014). To investigate the role of GABA in regulating oligodendrocyte development we used organotypic brain slices made from the frontal cortex of mice that express GFP in oligodendrocyte lineage cells (see Materials and Methods). Because the slices are able to retain the cyto‐architecture found in the cortex for many weeks, they are a good model to study neuron‐glial communication while allowing pharmacological manipulation. Fixing slices after different durations in culture, and labeling for neurofilament 200 (NF) and myelin basic protein (MBP), allowed us to image neuronal processes, oligodendrocyte lineage cells and myelin, and to monitor the development of compact myelin (Fig. 1).

**Figure 2:**
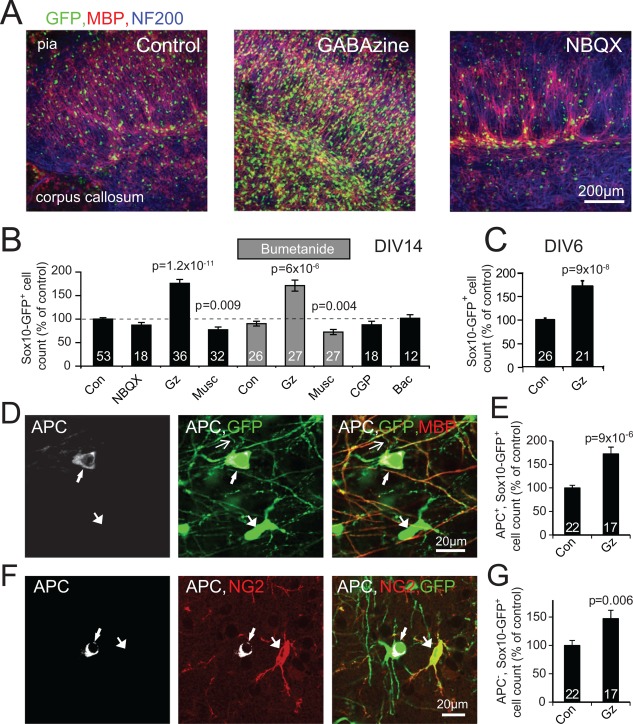
Blocking GABA_A_Rs increases the number of oligodendrocyte lineage cells. **A**: Cortical slices at DIV14 after culture in control conditions, or with GABA_A_ or AMPA/KA receptors blocked with GABAzine or NBQX respectively. **B**: Number of Sox10‐GFP‐expressing cells, in control conditions and with the indicated drugs present from DIV3‐14 (Gz, GABAzine; Musc, muscimol; CGP, CGP35348; Bac, baclofen). *P* values are Holm‐Bonferroni corrected for 5 comparisons for Gz, Musc, Bumetanide, CGP, Bac, and for 2 comparisons when comparing Bumetanide+Gz or Bumetanide+Musc with Bumetanide (numbers on bars are total numbers of slices from 12 experiments using two or three animals each). **C**: Effect of GABAzine from DIV3‐6 on number of GFP‐expressing cells at DIV6 (numbers on bars are total numbers of slices from 7 experiments using two or three animals each). **D**: Labeling of oligodendrocyte lineage cells (expressing GFP, green) for the mature oligodendrocyte marker APC (white) and for MBP (red). Top arrowed GFP‐expressing cell expresses APC, as well as MBP in its myelinating processes (top thin arrow indicates a primary process linking the arrowed soma to a MBP expressing process), unlike the bottom cell. **E**: Number of cells expressing GFP and APC in the presence of GABAzine, normalized to the number in control conditions (numbers on bars are total number of slices from 3 experiments using 3 animals each). **F**: Labeling of oligodendrocyte lineage cells (expressing GFP, green) for APC (white) and for the OPC marker NG2 (red). Unlike the left cell which expresses APC, the right arrowed cell lacks APC and expresses NG2. **G**: Number of cells expressing GFP but not APC in the presence of GABAzine, normalised to the number in control conditions (numbers on bars are as in E).

To test the effect of endogenously released glutamate and GABA on the number of oligodendrocyte lineage cells generated, we included the NMDA receptor blocker MK‐801 (50 μM), the AMPA/KA receptor blocker NBQX (25 μM) or the GABA_A_ receptor blocker GABAzine (50 μM; bicuculline was not used because it also blocks K^+^ channels: Seutin and Johnson, 1999) in the culture medium from days 3 to 14 in vitro. Blocking NMDA receptors led to a 40 ± 12% decrease (*P* = 0.02) of the amount of neurofilament labeling in the cultures, probably because neurons need a basal level of NMDA receptor activation to survive (Hardingham and Bading, 2003), which precluded a meaningful analysis of whether NMDA receptors regulate myelination. Deleterious effects on neurons were not observed when blocking AMPA/KA or GABA_A_ receptors. NBQX, which blocks excitatory synaptic transmission from axons to OPCs (Kukley et al., 2007; Ziskin et al., 2007), had no effect on the number of oligodendrocyte lineage cells present at DIV14 (Fig. 2A,B), nor on the amount of labeling for neurofilament (reduced by 3 ± 11%, *P* = 0.81) or MBP (increased by 4 ± 6%, *P* = 0.75). In contrast GABAzine, which blocks inhibitory synaptic transmission, dramatically increased the number of oligodendrocyte lineage cells (Fig. 2A,B); the increase over 7 independent sets of cultures (each from 2‐3 animals) was 1.76 ± 0.08 fold (Fig. 2B), implying that endogenous GABA release normally decreases the number of oligodendrocyte lineage cells by a factor of 1/1.76 or 43%.

The larger population of oligodendrocyte lineage cells observed in the presence of GABAzine included more mature oligodendrocytes. Using the combination of Sox10‐GFP expression and adenomatous polyposis coli (APC) antibody labeling to define mature oligodendrocytes (Bhat et al., 1996) we found that, by 2 weeks in culture, GABAzine produced a large fractional increase in the number of mature (APC‐expressing) oligodendrocytes (increased by 73%) as well as in the number of APC‐negative GFP‐positive OPCs (increased by 48%, Fig. 2D–G). Thus, endogenous GABA, acting via GABA_A_ receptors, greatly decreases the number of both OPCs and mature oligodendrocytes. Increasing GABA_A_ receptor activation, by applying muscimol (10 μM) (Yuan et al., 1998), decreased the number of oligodendrocyte lineage cells present at DIV14 by 23 ± 6% (Fig. 2B). Thus, increases and decreases of GABA_A_ receptor activation bidirectionally alter the number of oligodendrocyte lineage cells.

The increase of the number of oligodendrocyte lineage cells produced by GABAzine did not appear to depend on NKCC1 transporters accumulating Cl^‐^ in the cell to shift positive the reversal potential for GABA_A_ receptors (so that GABA depolarizes OPCs: Lin and Bergles, 2004; Tyzio et al., 2011). Blocking NKCC1 with bumetanide (100 μM, from 3 to 14 days in vitro) had no effect on the change of number of oligodendrocyte lineage cells produced by GABAzine or muscimol (Fig. 2B). Blocking GABA_B_ receptors with 50 μM CGP35348 or activating them with 10 μM baclofen also had no effect on the number of oligodendrocyte lineage cells present (Fig. 2B), unlike a previous report for pure OPC cultures (Luyt et al., 2007).

### GABA Regulates the Proliferation and Death of Oligodendrocyte Lineage Cells

GABAzine could increase the number of oligodendrocyte lineage cells either by blocking a GABA‐mediated suppression of OPC proliferation, or by blocking GABA‐evoked cell death. To investigate this we applied GABAzine from 3‐6 days in vitro, i.e. the period in Fig. 1E when OPC proliferation dominates. At day 6, NG2‐expressing OPCs were the majority of the oligodendrocyte lineage cells (68.5 ± 2.8% in control slices and 74.6 ± 2.4% in GABAzine treated slices, not significantly different, *P* = 0.11). By DIV6, GABAzine had evoked an increase in the number of oligodendrocyte lineage cells (Fig. 2C) similar to that seen at DIV 14 (Fig. 2B), and applying GABAzine after P6 had much smaller effects (data not shown) demonstrating that the effects of GABA_A_ signaling are exerted mainly at the OPC/early oligodendrocyte stage of development.

**Figure 3:**
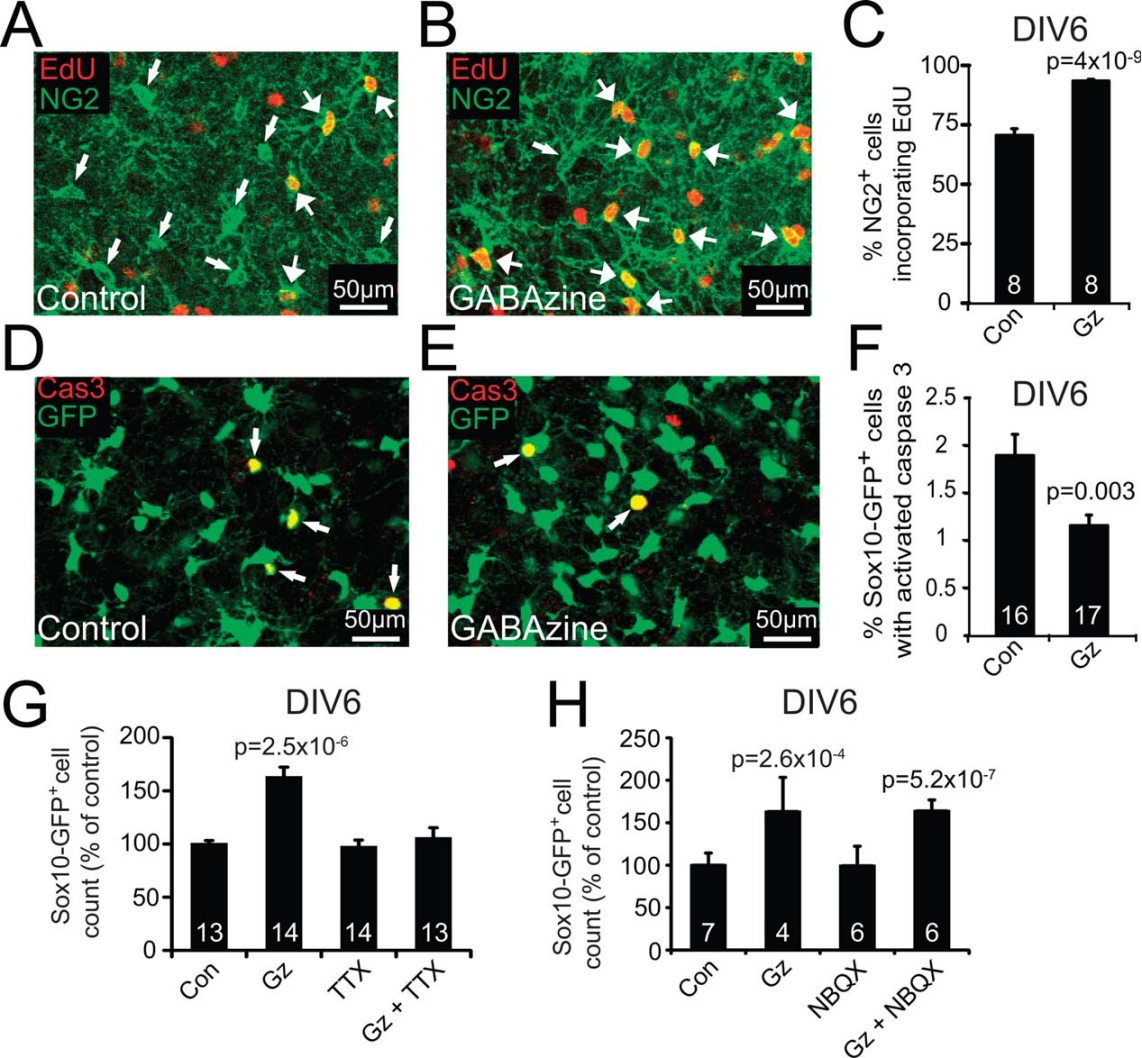
Endogenous GABA release decreases proliferation and increases death of oligodendrocyte lineage cells. **A,B**: Example of EDU labeling (red) in NG2 expressing cells (green) without (**A**) and with (**B**) GABAzine. **C**: Quantification of fraction of OPCs dividing between DIV4‐6 (numbers on bars are total number of slices from 2 experiments, each using 3 animals). **D‐E**: Example of cleaved caspase 3 labeling (red) in SOX10‐GFP expressing cells (green) without (**D**) and with (**E**) GABAzine. **F**: Quantification of fraction of oligodendrocyte lineage cells labeling for cleaved caspase 3 at DIV6 (numbers on bars are total number of slices from 2 experiments each using 3 animals). **G**: TTX has no effect alone on the number of oligodendrocyte lineage cells, but blocks its increase by GABAzine (numbers on bars are number of slices from 3 experiments each using 3 animals). **H**: NBQX does not affect the number of oligodendrocyte lineage cells nor its increase by GABAzine (numbers on bars are total number of slices from 2 experiments each using 2 animals).

Using EdU, we found that GABAzine increased the fraction of OPCs that were dividing between days 4 and 6 *in vitro* (Fig. 3A–C). In addition, labeling for apoptotic cell death at day 6 in vitro with antibody to cleaved caspase‐3, showed that GABAzine reduced the proportion of SOX10‐GFP cells undergoing apoptosis (Fig. 3D–F). Thus, endogenous GABA release normally suppresses OPC proliferation and increases cell death.

We investigated whether these effects of GABA were directly on OPCs, or mediated by changes of neuronal firing, or involved effects both on OPCs and on neurons. A direct suppressive effect of GABA on the proliferation of neural stem cells, mediated by a PI3K‐related kinase (PIKK) and histone H2AX, has been reported (Fernando et al., 2011). If GABA acts in the same way on OPCs then blocking the action of PIKK should mimic the effect of GABAzine in our experiments. However, we found that block of PIKK (using ATM kinase inhibitor, 10 μM) had no effect on the number of oligodendrocyte lineage cells (decreased by 3 ± 3% in 3 experiments using a total of 22 slices for each condition, not significant: *P* = 0.43).

Blocking neuronal firing by applying 1 μM TTX from Days 3 to 6 in vitro also had no effect on the number of OPCs produced (decreased by 2.9%, not significant, *P* = 0.7), but blocked the increase in OPC number produced by GABAzine (Fig. 3G, increased by 8.4%, not significant, *P* = 0.61). If GABA affects proliferation and cell death solely by acting directly on OPCs, this block could be explained by TTX suppressing action potential evoked release of GABA onto OPCs, or blocking voltage‐gated Na^+^ channels in OPCs that are activated by the depolarization (Lin and Bergles, 2004) that GABA produces. However, in both these scenarios, TTX alone should produce the same increase in proliferation as GABAzine, but it did not (Fig. 3G). An alternative hypothesis is that the increase in OPC number evoked by GABAzine, and its block by TTX, may be due to GABAzine increasing neuronal action potential firing, leading to the release from neurons of a factor promoting OPC proliferation and decreasing OPC death. However, although the GABA_A_ agonist muscimol (which should reduce neuronal firing) decreased the number of oligodendrocyte lineage cells (Fig. 2B), TTX (which should abolish firing completely) had no effect (Fig. 3G). These results might, however, be explained if there are two effects regulating OPC proliferation: a direct effect of GABA on OPCs which suppresses proliferation (Zonouzi et al., 2015), and release of a factor by active neurons that induces the expression of GABA_A_ receptors or signaling molecules downstream from them, that enables the number of oligodendrocyte lineage cells to be regulated by GABA (see Discussion).

In order to determine whether neuronal activity was needed to maintain GABA_A_ receptor expression in OPCs (cf. Arellano et al. (2016) who found that the presence of neurons is needed, but that activity is not), we applied TTX to the organotypic slices. We measured GABA‐evoked currents at −64 mV (with E_Cl_=‐4 mV) in patch‐clamped Sox10‐GFP‐expressing OPCs after incubation in TTX from DIV3 to DIV6, and found that the currents were not significantly reduced (19% decrease, *P* = 0.53, *n* = 9 with and *n* = 9 without TTX), in agreement with the lack of dependence of expression on neuronal spiking seen by Arellano et al. (2016).

We attempted to determine whether GABA has direct effects on OPC proliferation by making cortical OPC cultures and activating GABA_A_ receptors with muscimol. In accordance with a recent publication from the Matute laboratory (Arellano et al., 2016), GABA_A_ receptor mRNA expression was downregulated (by 83% and 78% respectively) for the GABA_A_ receptor α_1_ and β_2_ subunits in pure OPC cultures without the presence of neurons or neuronal‐conditioned media (after 10 days in mixed glial culture: see Methods). However, a GABA_A_ receptor mediated current was still observed in these cells (at DIV3 in pure OPC cultures after 10 days in mixed glial culture: see Methods). An outward current of 38 ± 8 pA was evoked by 100 μM GABA at −44 mV with E_Cl_ set to −87 mV, implying that GABA_A_ receptors were still functional in the cells. We found that activating GABA_A_ receptors with muscimol (10 μM), blocking them with GABAzine (50 μM) or applying TTX (1 μM), from DIV1 to DIV3 in pure OPC culture, had no effect on the number of proliferating cells, generating a 6.5% increase (*P* = 0.41), a 7.6% increase (*P* = 0.46), and a 3.5% decrease (*P* = 0.98), respectively.

Activation of voltage‐gated Na^+^ channels in OPCs by the depolarization produced by glutamatergic excitatory synaptic input to OPCs does not contribute to the GABAzine‐evoked increase in OPC number, because having NBQX present with the GABAzine did not prevent the increase in cell number produced by GABAzine (Fig. 3H).

### Endogenous GABA Release Decreases Myelination

**Figure 4:**
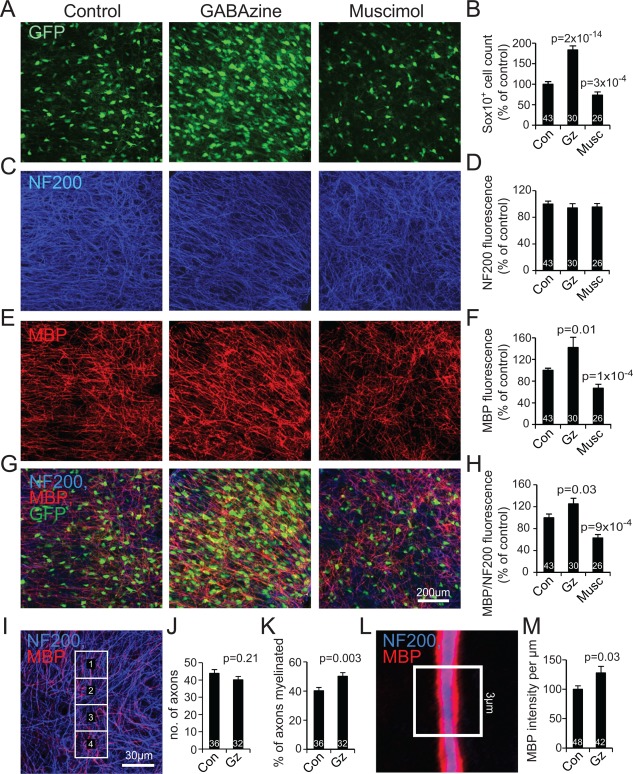
Endogenous GABA release decreases myelination. **A**, **C**, **E**, **G**: Cortical slices at DIV14 after culture in control conditions, or with GABAzine or muscimol present from DIV3‐14, showing labeling for (**A**) Sox10‐GFP, (**C**) neurofilament (NF200), (**E**) MBP and (**G**) all the labels. **B**, **D**, **F**: Quantification (averaged over the whole image) of the labeling in the panels to the left (panel B is similar to the 1st, 3rd, and 4th bars in Fig. 2B but using data only for the slices from which panels D, F, and H were obtained; numbers on bars are total number of slices from 7 experiments using 2 or 3 animals each). **H**: Ratio of labeling for MBP to that for NF200. **I**: Specimen labeling with superimposed squares for quantification of the fraction of NF200‐expressing axons (blue) that are myelinated (i.e., wrapped with MBP, red). **J‐K**: Effect of GABAzine from DIV3‐14 on the number of axons present per rectangle (**J**) and the percentage of axons myelinated (**K**); numbers are total number of areas from 8 slices, in 2 experiments with 3 mice each. **L**: Specimen image of myelinated axon, with 3 μm long region of interest used to quantify the amount of MBP per micron of axon. **M**: Effect of GABAzine on MBP fluorescence intensity per micron of axon. Numbers on bars are total number of axons in 8 slices, from 2 experiments using 3 mice each.

In contrast to the effect of GABAzine and muscimol on oligodendrocyte lineage cell number, neurofilament labeling was unaffected by these drugs, implying little effect on the growth of neuronal processes, while total MBP fluorescence was increased by 42 ± 19% by GABAzine and decreased by 33 ± 7% by muscimol (Fig. 4A–F). As an index of myelination per neuronal process, we normalized the summed fluorescence of the MBP present to the summed fluorescence of the neurofilament present. GABAzine increased this index by 26 ± 10%, while muscimol decreased it by 37 ± 6% (Fig. 4G,H). The GABAzine‐evoked myelination increase was, in part, the result of GABAzine increasing by 25 ± 6% the fraction of axons myelinated, without significantly changing the number of axons present (Fig. 4I‐K). This implies that endogenous GABA release normally decreases the fraction of axons myelinated by a factor of 1/1.25 or 20%.

By selecting only myelinated axons, and measuring the MBP fluorescence per length of axon (Fig. 4L), we found that GABAzine also increased the amount of myelin labeling per length of axon by 28 ± 11% (Fig. 4M), implying that endogenous GABA release normally decreases the amount of myelin per myelinated axon by a factor of 1/1.28 or 22%. This might reflect an increase in myelin thickness, or in axon diameter (measurement of which is inaccurate in our light microscopy images), or both. Thus, endogenous GABA release normally decreases both the fraction of axons myelinated and the amount of myelin per axon.

### Endogenous GABA Increases Internode Length

**Figure 5:**
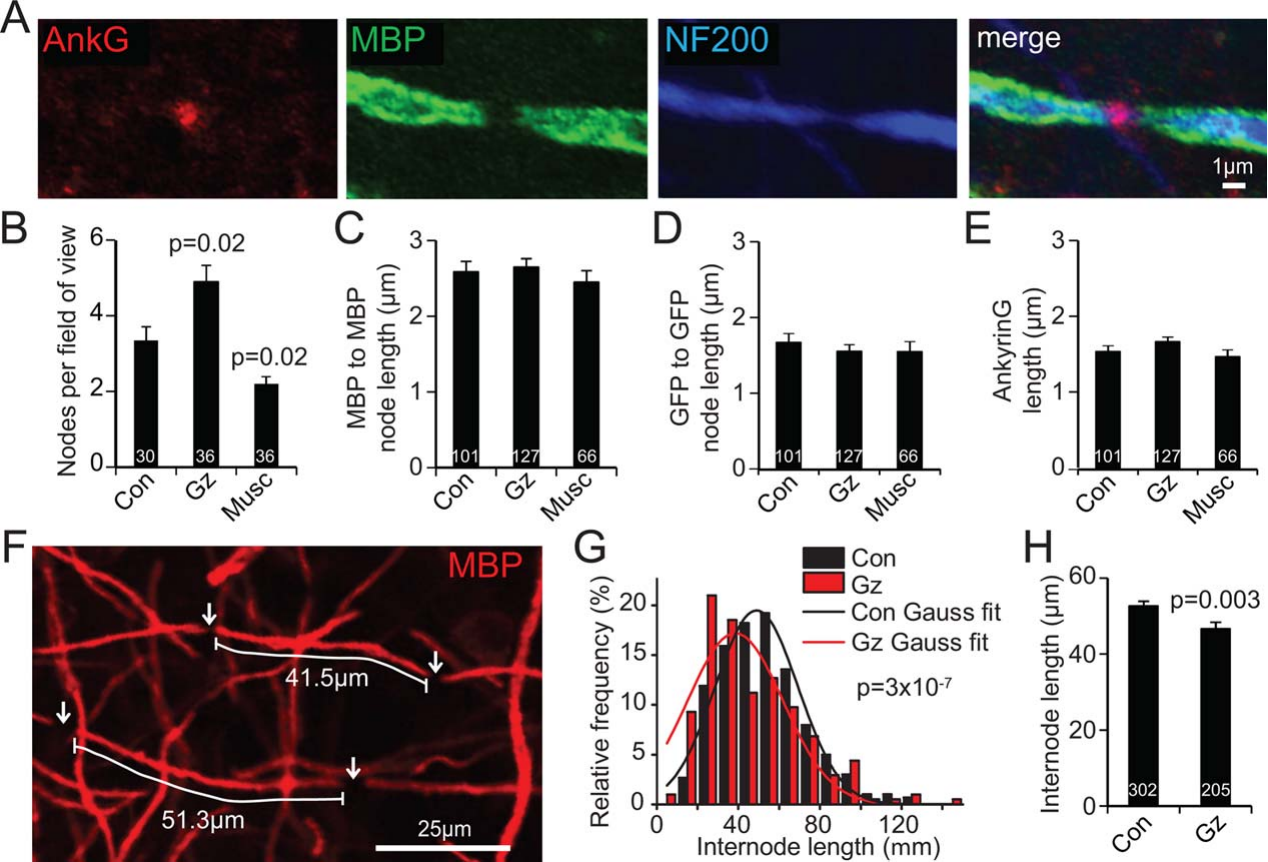
Endogenous GABA release increases internode length. **A**: Myelinated axon node of Ranvier labeled for ankyrin G (Ank G), myelin basic protein (MBP) and neurofilament (NF200); right panel shows merged image. **B**: Nodes per 146 μm square field of view at DIV14, in control conditions, or with GABAzine or muscimol present from DIV3‐14. Numbers on bars are fields of view; 6‐10 fields were taken per slice (a total of 6 slices were used from 2 mice). **C‐E**: Node lengths (measured from all the nodes imaged for panel B) in different conditions assessed as: (**C**) gap between end of internodal MBP labeling, (**D**) gap between internodal GFP, and (**E**) length of ankyrin G labeling. Numbers on bars are total numbers of nodes counted in B. **F**: Example of internode length measurement. Nodes are identified as gaps in GFP and MBP labeling. **G**: Distribution of internode lengths, L (in 10 μm bins), at DIV14, in control conditions (in eight 92 μm x 160μm images from 4 slices taken from 2 animals), or with GABAzine present from DIV3‐14 (7 images from 4 slices). Fits are Gaussian curves, {*A*/[*σ*√(2*π*)]}exp{‐(L‐M)^2^/(2*σ* ^2^)}, with parameters *A* = 991, mean length *M* = 48.9 μm, *σ* = 20.3 μm in control and *A* = 1004, *M* = 38.5 μm, *σ* = 23.3 μm in GABAzine. *P* value showing significantly different distributions is from Kolmogorov‐Smirnov test. H, Mean internode length from (G); numbers on bars are internodes.

The myelin sheath internode length has previously been postulated to be set solely by the axon diameter (Rushton, 1951) or to be an intrinsic property of the oligodendrocytes carrying out the myelination (Bechler et al., 2015). However, in GABAzine there are more OPCs competing to myelinate the same number of axons. In addition to this being a possible reason why more axons become myelinated, it may result in each oligodendrocyte making shorter internodes. We identified Ranvier nodes using antibody to ankyrin G, or as gaps in the MBP‐ and GFP‐labeling of the myelinating processes of oligodendrocytes (Fig. 5A). GABAzine increased, while muscimol decreased, the number of nodes in each 146 μm square field of view (Fig. 5B). The 47% increase in node density produced by GABAzine is larger than the 25% increase in the number of axons myelinated (Fig. 4K), implying that GABAzine decreases the separation of nodes along axons, i.e. decreases the internode length. GABAzine and muscimol did not alter the lengths of the nodes themselves (Fig. 5C–E). The distribution of internode lengths differed significantly between control slices and GABAzine‐exposed slices (Fig. 5F–G), corresponding to a 13% decrease of mean internode length in GABAzine treated slices (Fig. 5H: the Gaussian fits to the distributions in Fig. 5G predict a 21% decrease), which implies that endogenous GABA release normally increases the mean internode length by a factor of 1/1.13 or 12%.

## Discussion

Our data reveal that the neurotransmitter GABA exercises a major influence on the number of oligodendrocyte lineage cells in situ in cerebral cortical slices. Blocking the effects of GABA on GABA_A_ receptors nearly doubled the number of oligodendrocyte lineage cells (Fig. 2A,B), increased myelination (Fig. 4G,H) and decreased internode length (Fig. 5G,H). Thus, endogenous GABA release is important in determining the development of these cells (Zonouzi et al., 2015).

In contrast, blocking endogenous glutamatergic excitation had no effect on the number of oligodendrocyte lineage cells (Fig. 2B). These observations are in contrast to those of Yuan et al. (1998), who found that glutamate decreased the number of oligodendrocytes in cerebellar slices, while GABA had no effect. The reason for this difference is unclear (although we note that LoTurco et al. (1995) found a suppressive effect of GABA on cortical progenitor cell proliferation, similar to what we find for oligodendrocyte lineage cells). It may imply a difference between the neocortex and the cerebellar cortex in the mechanisms regulating cell proliferation and myelination.

The increase in the number of CC1‐expressing mature oligodendrocytes and myelination seen in the presence of GABAzine contrasts with the inhibition of lineage progression reported by Zonouzi et al. (2015) when the GABA_A_ receptor blocker bicuculline was injected in vivo. This could reflect the fact that bicuculline salts have nonspecific actions and, in addition to blocking GABA_A_ receptors, also block Ca^2+^‐activated K^+^ channels (Seutin and Johnson, 1999). The increase of myelination is presumably a result of the increase in the number of oligodendrocyte lineage cells which is produced (Fig. 3A–F) by an increase of proliferation of OPCs, and also a decrease of cell death (which occurs both at the OPC stage and after differentiation into pre‐myelinating oligodendrocytes: Barres et al., 1992; Trapp et al., 1997). The increased number of OPCs available for myelination (Fig. 2B,C,G) increases the fraction of axons that become myelinated (Fig. 4K) and decreases internode length, presumably because more OPCs myelinate the same axon (Fig. 5F–H). Thus, the number of oligodendrocyte lineage cells that are produced and survive—and the resulting myelination—are determined not only by a cell‐intrinsic clock and/or the availability of growth factors (Raff et al., 1988; Calver et al., 1998; van Heyningen et al., 2001), but also by locally released GABA.

How GABAergic signaling suppresses proliferation and increases cell death remains unclear. We have shown that it does not depend on the reversal potential for GABA‐evoked currents being maintained positive to the resting potential by NKCC1, since the effect of GABAzine on oligodendrocyte lineage cell number was unaffected by blocking NKCC1 with bumetanide (Fig. 2B), and so is unlikely to reflect GABA‐evoked depolarization initiating a Ca^2+^ influx through voltage‐gated calcium channels (although we cannot rule out the possibility that another mechanism keeps [Cl^‐^]_i_ high). In contrast, Zonouzi et al. (2015) observed that knock‐out of NKCC1 had effects similar to the application of bicuculline. We considered the following hypotheses for how GABAzine might increase the number of oligodendrocyte lineage cells.

First we postulated that GABA regulates proliferation solely by acting on oligodendrocyte lineage cells (as suggested by Zonouzi et al., 2015). The increase of OPC number produced by GABAzine (Fig. 2B), would then imply that there is a tonic release of endogenous GABA onto OPCs. The lack of effect of TTX on proliferation (Fig. 3G) would imply that this GABA release is not action potential driven. On this hypothesis, therefore, the GABA would have to be released in an action potential independent manner, perhaps from OPCs or astrocytes, as both synthesize GABA from putrescine using monoamine oxidase B (Barres et al., 1990; Yoon et al., 2014). Inconsistent with this, however, we found that GABAzine had no effect on proliferation in the presence of TTX (Fig. 3G).

We therefore turned to the idea that all of the actions of GABAzine are solely on neurons, with GABAzine increasing spiking and promoting the release of a substance that promotes OPC proliferation. This would be consistent with GABAzine having no effect in TTX (Fig. 3G), but to explain why TTX alone has no effect (Fig. 3G) we would have to also postulate that the spiking rate is very low in the absence of GABAzine. If this were the case, however, the GABA_A_ agonist muscimol (which is expected to decrease the spiking rate like TTX), should also have no effect on OPC proliferation, but in fact it decreases proliferation and myelination (Figs. 2B and 4F).

Thus, the effects of the GABAergic agents and TTX that we have observed apparently cannot be explained solely in terms of an action of GABA solely on OPCs (as postulated by Zonouzi et al., 2015) or solely on neuronal spiking. However, the combination of spiking‐induced release of a factor regulating OPC proliferation and a direct suppressive effect of GABA_A_ receptor activation on OPC proliferation might explain the results. The difference in behaviour from the cerebellar OPCs studied by Zonouzi et al. (2015) could reflect the difference of brain area studied.

While the exact mechanisms remain to be defined, it is clear that the development of grey matter OPCs is locally regulated by GABA release (presumably from nearby interneurons: Mangin et al., 2008), which decreases myelination but increases internode length (Figs. 4 and 5). Consequently, interneuron activity can tune the conduction speed of nearby axons, and differences in the local density or activity of interneurons along the path of a set of axons might lead to a spatial variation (Tomassy et al., 2014) of the fraction of axons that become myelinated, or of the internode length in the axons that do become myelinated. A spatial variation of internode length has been reported for axons in the auditory system and is predicted to affect conduction speed (Brill et al., 1977; Ford et al., 2015; Seidl et al., 2010).

Finally, as suggested by recent findings (Zonouzi et al., 2015), it is likely that GABAergic effects on myelination could play a role in pathology. Therapeutic drugs affecting the activity of GABA_A_ receptors might alter CNS myelination if they are administered during the period over which oligodendrocytes develop. Indeed, in rats, the anti‐epileptic GABA_A_ agonist phenobarbital decreases myelin formation in rat pups when administered either to the pups or to their mothers before birth (Patsalos and Wiggins, 1982).
